# Recent Advances in the Wearable Devices for Monitoring and Management of Heart Failure

**DOI:** 10.31083/j.rcm2510386

**Published:** 2024-10-28

**Authors:** Victor Adeyi Odeh, Yifan Chen, Wenyan Wang, Xiaorong Ding

**Affiliations:** ^1^Department of Biomedical Engineering, School of Life Science and Technology, University of Electronic Science and Technology of China, 610054 Chengdu, Sichuan, China; ^2^Heart Failure Center, Sichuan Academy of Medical Science and Sichuan Provincial People’s Hospital, University of Electronic Science and Technology of China, 610054 Chengdu, Sichuan, China

**Keywords:** heart failure, wearable devices, vital signs, physical activity, cardiovascular monitoring

## Abstract

Heart failure (HF) is an acute and degenerative condition with high morbidity and mortality rates. Early diagnosis and treatment of HF can significantly enhance patient outcomes through admission and readmission reduction and improve quality of life. Being a progressive condition, the continuous monitoring of vital signs and symptoms of HF patients to identify any deterioration and to customize treatment regimens can be beneficial to the management of this disease. Recent breakthroughs in wearable technology have revolutionized the landscape of HF management. Despite the potential benefits, the integration of wearable devices into HF management requires careful consideration of technical, clinical, and ethical challenges, such as performance, regulatory requirements and data privacy. This review summarizes the current evidence on the role of wearable devices in heart failure monitoring and management, and discusses the challenges and opportunities in the field.

## 1. Introduction 

Heart failure (HF) is a persistent and incurable clinical condition brought on by either inadequate myocardial relaxation, decreased ejection, or a combination of the two. Many disorders, such as coronary artery disease, hypertension, atrial fibrillation, heart valve disorders, excessive alcohol consumption, infections, cardiomyopathy with unknown causes, and structural abnormalities of the heart can result in HF [1, 2]. It is important to note that the presentation of these symptoms can vary from person to person, and their severity often depends on the individual and the stage of the condition. Common indications and symptoms of HF include shortness of breath, fatigue, muscle weakness, swelling in the lower extremities (legs, ankles, or feet), an irregular or rapid heartbeat, persistent coughing or wheezing, and difficulty sleeping due to breathing difficulties [3]. HF is a global health issue, particularly in developed countries [4]. In the US, 6.2 million adults suffer from it, with a projected 46% increase by 2030 [5]. Factors include aging population, chronic disease management, acute coronary syndrome treatments, and improved care [6]. Europe faces 15 million cases, resulting in over 3 million hospitalizations annually. High prevalence and re-hospitalization rates cause significant economic burdens on healthcare systems and society [4].

Wearable technology, commonly referred to as “wearables”, encompasses electronic devices that are designed to be worn on the body. These devices can take various forms, including accessories, clothing, medical devices, and even items that can be implanted in the body or tattooed on the skin. Wearables are often equipped with microprocessors, sensors, and connectivity features, allowing them to collect data, track activities, and communicate with other devices such as smartphones or computers [5, 6]. Wearables are a significant part of the Internet of Things (IoT) industry, contributing to its growth by allowing users to stay connected and informed about their personal data in real time.

Recent breakthroughs in wearables have ushered in a new era in the management of HF, transforming the landscape of cardiovascular care. These technological advancements go beyond conventional monitoring approaches, offering innovative solutions that empower patients and provide clinicians with real-time insights. Wearable devices, equipped with an array of sensors and sophisticated algorithms, now play a pivotal role in tracking vital signs, detecting anomalies, and promoting proactive healthcare.

This article provides a comprehensive review of recent advances and the most recent evidence regarding the significance of wearables in the detection, diagnosis, and management of HF. Given the complexity of HF as a disease state, this review focuses on the specific contributions of wearable technology that directly impact patient management. It avoids overly general information about heart disease that does not directly relate to wearable technology’s impact, ensuring that each point made is relevant and directly supports the narrative of technological advancement and patient-centric care. In Section II, we present the roles and functions of wearable technologies that are for HF monitoring and management, Table 1 (Ref. [7, 8, 9, 10, 11, 12, 13, 14, 15, 16, 17, 18, 19, 20, 21, 22, 23, 24, 25, 26, 27, 28, 29, 30, 31, 32, 33, 34, 35, 36]) also gives a summary of wearables used in HF management. We also looked at stealthy sensor innovations that can be applied to widespread home and public monitoring. The final section of this article will address current limitations, collection of the challenges these initiatives face or factors that lead to initiative failures.

**Table 1.** **Summary of Wearables in Heart Failure Management**.

Function	Study	Implication on heart failure	Wearable device	Monitoring indicators	Samples (N)	Findings
Continuous monitoring of blood oxygen saturation (SpO_2_).	Research has shown that low SpO_2_ levels is linked to higher mortality rates in individuals suffering from HF [11, 12, 13].	Studies found that changes in SpO_2_ levels were a reliable early indicator of impending HF decompensation and the pulse oximetry significantly impacts the diagnosis and severity of HF in patients with acute myocardial infarction, potentially impacting prognosis and reducing mortality rates [14, 17].	Timesco CN130, Loop (SpryHealth, CA, USA) [17]	Offers an affordable, reliable, and accurate way to check pulse and SpO_2_ levels, cleared by the FDA and has an accuracy of +99%.	220	Pulse oximetry significantly impacts the diagnosis and severity of HF in patients with acute myocardial infarction, potentially impacting prognosis and reducing mortality rates [15].
			Oxitone 1000M [16]	Measures SpO_2_, respiratory rate, pulse rate, cleared by the FDA and has an accuracy of 97% (for SpO_2_).	12	Monitoring of vital signs and activity levels and improved detection of arrhythmias and abnormalities [16].
			Biostrap (Biostrap, CA, USA), [30]	Provides biometric information, such as HR and deep sleep through a clinical grade pulse oximeter.	78	A study demonstrates that the addition of a well-known standalone PPG-AF detection algorithm to a Biostrap wristband yields a high accuracy for the detection of AF, with an acceptable unclassifiable rate, in a semi-controlled environment [30].
Monitoring of heart rate and activity.	Individuals with HF commonly have elevated resting heart rates, which are associated with worse clinical outcomes, such as an increased risk of hospitalization, morbidity, and mortality [7].	Continuous monitoring of heart rate, rhythm, and ECG data aids in early identification of HF exacerbation symptoms, enhancing patient outcomes and the management of HF may benefit from heart rate monitoring, according to the authors, as it can help pinpoint individuals who are most at risk for negative outcomes and provide useful information on the course of the disease [7, 8].	VitalPatch [9]	Evaluates heart performance, sends patient information to a secure cloud for real-time cardiac arrhythmia monitoring.	2659	A study found that individuals monitored by this means had an opportunity to receive care earlier if AF was detected, if compared with unmonitored controls [32].
			BodyGuardian® Heart [23]	Small wireless heart activity monitor that adheres to the chest via a disposable strip. The strip can be repositioned as needed thanks to its medical-grade adhesive and electrode gel and should be replaced periodically during the monitoring period.	12	The BodyGuardian device detected clear HR responses after amphetamine administration while subjects were physically active, whereas conventional measures collected at predefined timepoints while subjects were resting and supine did not [30].
			Huawei Band 6 [10]	Monitors HR 24/7, day and night SpO_2_. Tracks menstrual cycle, sleep, and stress.	106	Huawei smart wearables have been shown to have a high positive predictive value for detecting AF in the general population [31, 33].
			ZioXT [10]	The patch records ECG data for up to 14 days, detecting irregular heart rhythms like arrhythmia, and its data is sent to a treating physician for analysis. It has a 99% accuracy.	76	ZioXT was found to be more effective than a 24-Holter monitor in a study for the detection of AF, which led to an increase in clinical accuracy, the identification of potentially harmful arrhythmias, and a significant shift in clinical care [10].
Monitoring physical activity and exercise levels.	Reduced cardiovascular morbidity and mortality are linked to even moderate improvements in physical activity (PA), across the general population and in people with pre-existing heart conditions [20].	Physical activity may decrease the progression of the illness by lowering the prevalence of HF risk factors, promoting physiological cardiac remodeling, and enhancing mortality and HF symptoms in people who already have the condition [21].	Fibit smartwatch [24]	The system monitors patients’ exercise, sleep quality, breathing rate, skin temperature, energy expenditure, menstrual health, stress, moods, guided breathing sessions, heart rate, and cardiovascular fitness.	455,699	In the Fitbit Heart study, a novel software algorithm compatible with a wide range of smartwatches and fitness trackers detected irregular heart rhythms and accurately identified undiagnosed AF 98% of the time and this will prompt early HF care [34].
			Chest Strap [22]	Chest straps enable precise heart rate measurements, aiding in monitoring activity intensity and duration, and tracking progress towards goals.	220	The chest strap was used to detect arrhythmias in 220 patients, the study found that chest straps can be used for long-term follow-up to detect AF and this reduces readmission of patients by 38% [25].
			Apple Watch Series 6 [20]	Reads blood oxygen levels. Monitors HR and PA. Records sleep hours, among others.	200	This study showed 0.5% participants received irregular heart rhythm notification, the study showed that the apple watch can help with early identification of HF progression [35].
Remote monitoring and provision of real-time feedback.	These wearables can be linked to remote monitoring systems, giving medical professionals access to real-time information on a patient’s heart rate, physical activity, blood pressure, and other vital signs [18].	These wearables can be linked to remote monitoring systems, giving medical professionals access to real-time information on a patient’s heart rate, physical activity, blood pressure, and other vital signs. This may make it possible for medical professionals to spot changes in a patient’s condition before they worsen and to administer prompt therapies to stop HF from progressing [18].	Link HF [27]	Performs continuous monitoring of BP, blood oxygenation, track ECG, breathing rate, skin temperature and physical activity.	100	The platform detected precursors of hospitalization for HF exacerbation with 76% to 88% sensitivity [27].
	These devices for remote monitoring have proven to reduce re-hospitalization and have appeared feasible for HF medication escalation in HF patients [19].		Vital Patch [9]	Monitors cardiac function. Sends patient data to a secure cloud for real-time monitoring of different cardiac arrhythmias and has an accuracy of 59.2%.	65	Wearable biosensor was used to continuously remotely monitor patients with HF for 30 days after discharge shows it offers a low-risk solution to improve care of patients with HF after hospital discharge and may help to decrease readmission of patients with HF to the hospital and earlier detection of clinical deterioration [36].
			Vivometrics (The LifeShirt system) [26]	Records BP and HR to later send the records to a health professional for medical diagnosis.		
Early detection of decompensation.	A biomarker for pulmonary congestion and impending decompensation is intrathoracic impedance. Wearable technology is able to track variations in this impedance over time, which can be used to identify signs of acute events or worsening symptoms [25].	The need for immediate hospitalization of HF patients is a critical event, and in-hospital mortality rate ranges from 4 to 10% [25].	ReDS™[29]	Monitors Intrathoracic impedance which can be a biomarker for pulmonary congestion and impending decompensation.	50	Observational study of 50 patients to study HF readmission, it was found that HF readmissions at 3 months was reduced by 87% [29].
			ZOLL Cor™ (NCT03476187) [28]	Measures pulmonary fluid levels and has an ECG monitor, radiofrequency sensor, and transmitter is currently being tested in a clinical trial for its ability to foretell HF decompensation.	500	Trial demonstrated the impact of utilizing ZOLL HF Management System (HFMS) for fluid management following an acute decompensation event with 30% reduction in 90-day hospital readmission [28].

HF, heart failure; FDA, Food and Drug Administration; HR, heart rate; PPG-AF, photoplethysmography-atrial fibrillation; ECG, electrocardiographic; AF, atrial fibrillation; BP, blood pressure.

## 2. Wearables in Monitoring and Managing of Heart Failure

### 2.1 Overview

As the name suggests, wearables are electronic devices that can be attached to the body to track a variety of physiological functions, including blood pressure, heart rate, breathing rate, physical activity, blood sugar levels, and sleep patterns. Wristbands, eyeglasses, in-ear monitors, chest straps, and electronic garments are examples of wearables. With the help of cutting-edge technology, they were developed with the intention of making our lives simpler by giving us instant access to many types of data without the need for a separate device. “Wearable tech” was developed with the consumer in mind and is meant to promote beneficial behavioral changes [37].

It is impossible to overestimate the benefits and roles of wearables in HF management. By performing several significant roles and tasks, wearables ultimately aim to enhance outcomes, decrease re-hospitalization, and decrease morbidity and mortality rates [38]. Wearables can assist medical professionals in making better decisions about pharmaceutical therapy, exercise routines, and other facets of patient care by continuously monitoring and tracking physiological data. In order to further contribute to better outcomes, wearables can also help patients manage their own care and take their medications as prescribed. The many capabilities and features of wearable systems for monitoring and managing HF, addressing their selection, functionalities, and impact on patient outcomes will be covered in this review.

### 2.2 Selection of Wearables 

The selection of wearable devices is a decision influenced by a multitude of factors, with individuals carefully considering their specific needs, preferences, and objectives. A pivotal factor in choosing a wearable device is health and fitness goals. For instance, those aiming to monitor daily steps, track heart rate during workouts, or analyze sleep patterns often opt for fitness trackers or smartwatches equipped with health-monitoring features [39]. Additionally, data accuracy is another critical consideration, prompting users to research and select devices known for their precise sensors and reliable data measurements [40]. Compatibility with existing devices and ecosystems is often a deciding factor, as individuals seek seamless integration, with choices often influenced by brand loyalty [41]. Moreover, the design and aesthetics of wearables are significant to users, with preferences ranging from sleek, minimalist designs to rugged options suitable for outdoor activities [42]. Battery life can be a crucial factor, particularly for wearables intended for all-day use or extended activities [43]. Budget considerations weigh heavily in the decision-making process, as wearables are available at various price points, and individuals choose devices that align with their financial constraints [44]. Reviews, recommendations from friends or online communities, and consultations with healthcare professionals all play pivotal roles in the decision-making process [45].

**Fig. 1.**
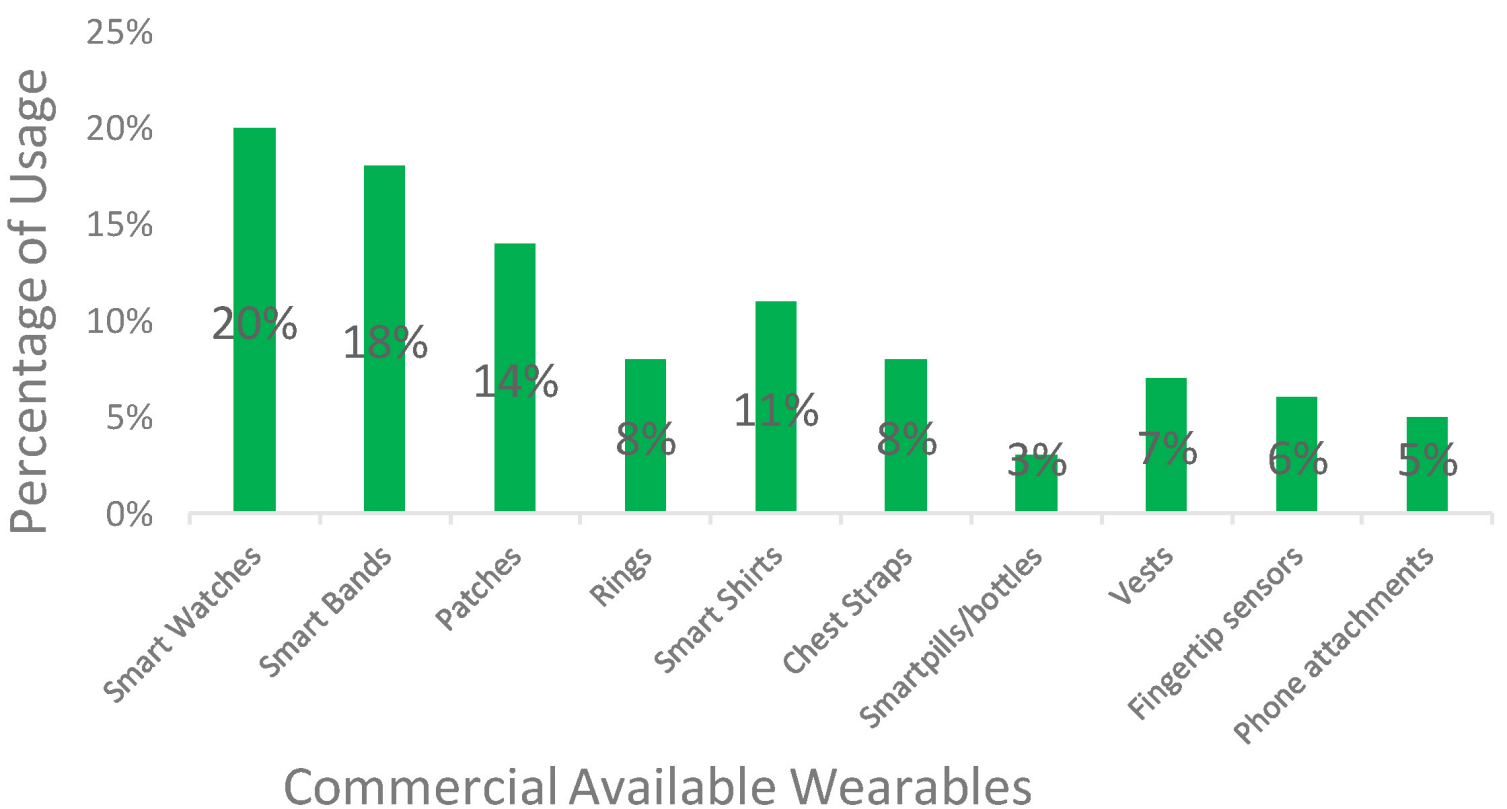
**Diverse landscape of technological solutions available for HF monitoring**. HF, heart failure.

Among the most prominent wearables used for HF monitoring are smartwatches and wristbands, along with mobile apps. Although less common, other wearables such as rings and wearable vests are also explored in the literature. Fig. 1 illustrates various wearables and their use in the management of HF. Notably, there’s a growing interest in sensor-based technologies, particularly those integrated into widely accepted wearables like wristbands and smartwatches. These devices are favored by users for their comfort, unobtrusiveness during daily activities, and ease of integration. Patches, although less frequently used in the literature, signify a rising trend in sensor-based technologies, showcasing the continual evolution of wearable devices for HF management. The figure below illustrates the types of wearable devices encompassed in the review, reflecting the diverse landscape of technological solutions available for HF monitoring.

### 2.3 Continuous Monitoring of Vital Signs

The management of HF requires continuous monitoring of vital signs and symptoms to detect changes in health status and adjust treatment plans accordingly. Regular vital sign monitoring is a typical inpatient care intervention that tries to make it easier to identify abnormal physiological parameters in patients who are deteriorating. A study by Iqbal *et al*. [46], revealed that the quality of life, effective care and management of HF patients can be improved by continuously advancing our knowledge of the basic pathophysiology of HF, increasing our ability to recognize high-risk patients, and enabling physicians customize therapies to an individual’s specific risk profile by careful monitoring of vital signs using wearable technology. Among the crucial vital signs monitored in HF are:

*(i) Continuous Blood Pressure Monitoring:* More than 10 million deaths that could have been prevented globally each year are caused by heart disease and hypertension, the major causes of morbidity and mortality [47]. Hypertension is a common risk factor for developing HF and can worsen the condition in patients who already have it [48, 49]. Wearables that can measure blood pressure continuously or at regular intervals can provide valuable data to healthcare providers, allowing them to adjust treatment plans accordingly and may help to mitigate unexpected aggravation, lower the risk of re-hospitalization or mortality, and prevent sudden deterioration.

**Fig. 2.**
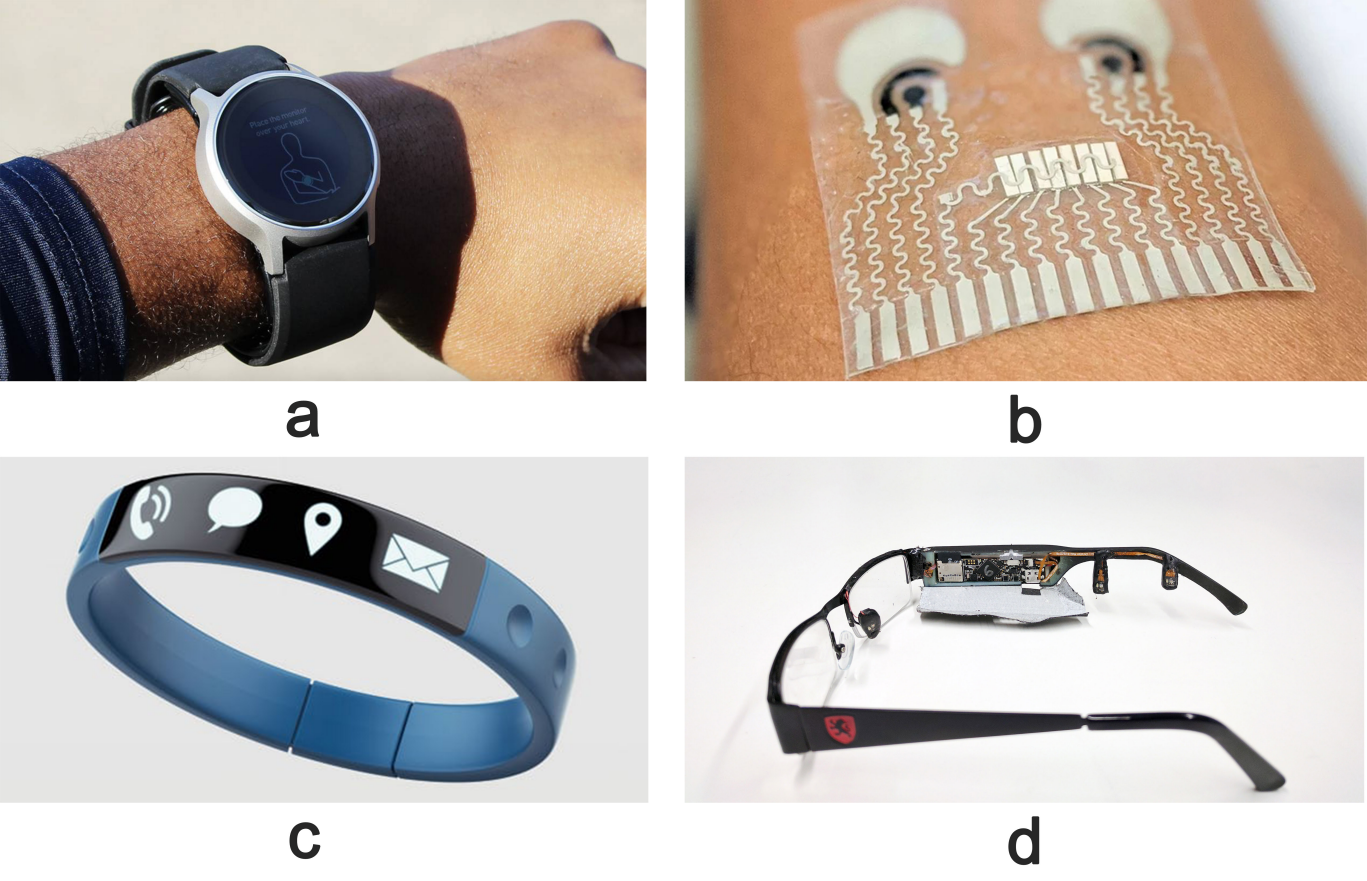
**Platforms for unobtrusive/wearable blood pressure monitoring that are realized in everyday items**. (a) BP watch. (b) Wearable skin-like BP patch. (c) Wrist/armband. (d) BP eyeglasses. BP, blood pressure.

Although an arterial invasive line can be utilized to readily deploy continuous blood pressure monitoring in intensive care units, various approaches have been proposed to overcome the limitations of traditional blood pressure measurement devices and measure blood pressure non-invasively with wearables such as smartwatches [50], wrist/armbands [51], sleeping cushions [52], chairs [53], smartphones [54], glasses or flexible patches [55]. Fig. 2 displays various non-invasive wearables used for the continuous monitoring of blood pressure. These wearables measure blood pressure utilizing several technologies, such as oscillometry and Photoplethysmography (PPG), which are easily linked with a miniaturized wireless unit for Blood Pressure (BP) mHealth monitoring suited for a variety of applications to support ongoing HF monitoring. Another commercially available option for monitoring HF is the Samsung Galaxy Watch Active2. Kim *et al*. [56], studied the accuracy and dependability of the Samsung Galaxy Watch Active2 for continuous blood pressure monitoring in patients with HF. According to the study, the gadget demonstrated good accuracy and reliability when compared to traditional blood pressure measurement techniques, pointing to its potential use in HF patients’ blood pressure monitoring [56]. Li *et al*. [57] developed an optical fiber sensor-assisted smartwatch for precise continuous blood pressure monitoring, achieving accurate measurements within acceptable ranges. Sel *et al*. [58], introduced ring-shaped bioimpedance sensors that leverage deep tissue sensing ability for continuous blood pressure estimation, showing high correlations and low errors. Min *et al*. [59] reported a wearable piezoelectric blood pressure sensor with high sensitivity and fast response time, demonstrating accurate measurements compared to a commercial sphygmomanometer. Zhou *et al*. [60], reviewed wearable continuous blood pressure monitoring devices based on the pulse wave transit time method, highlighting their advantages in terms of dynamic response characteristics and accuracy. These studies demonstrate the potential of wearables for continuous blood pressure monitoring, offering convenient and accurate solutions for cardiovascular health management.

*(ii) Heart Rate:* Heart rate is a key factor in HF diagnosis, prognosis, and treatment. In healthy individuals, the autonomic nervous system carefully regulates heart rate, which symbolizes the balance of sympathetic and parasympathetic activity. However, with HF, the autonomic nervous system is out of balance, leading to increased sympathetic function and decreased parasympathetic function. As a result, the heart’s capacity to respond to stress and exercise is hampered, heart rate variability is decreased, and resting heart rate is increased. Individuals with HF commonly have elevated resting heart rates, which are associated with worse clinical outcomes, such as an increased risk of hospitalization, morbidity, and mortality [7]. These findings originate from a study by Wang *et al*. [7], a sizable cohort of more than 5000 participants took part in the study. The management of HF may benefit from heart rate monitoring, according to the authors, as it can help pinpoint individuals who are most at risk for negative outcomes and provide useful information on the course of the disease.

**Fig. 3.**
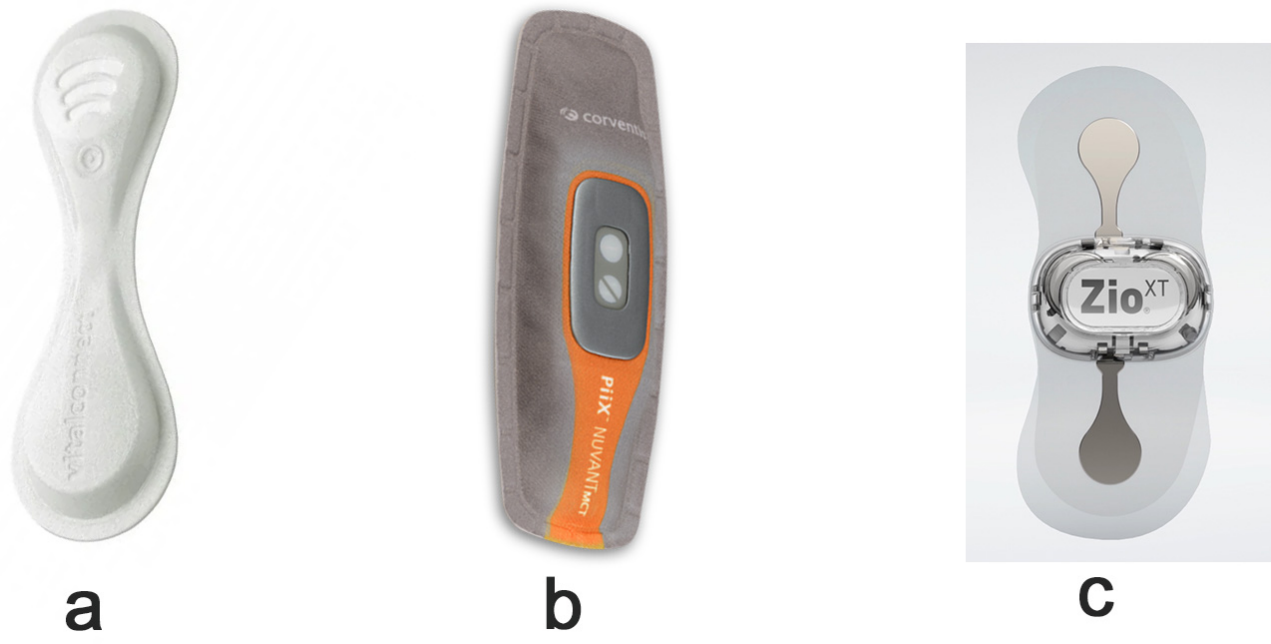
**Commercial ECG patches used in clinical trials and in the monitoring of heart rate in HF management**. (a) VitalPatch wearable sensor used in the continuous monitoring of heart rate in HF patients. (b) NUVANT (Corventis, San Jose, CA, USA) used for providing a convenient and reliable method for monitoring heart function. (c) The ZioXT (iRhythm, San Francisco, CA, USA) used for continuous monitoring of vital signs in elderly individuals to detect and prevent deterioration of patients with HF. ECG, electrocardiographic; HF, heart failure.

In order to prevent a condition from getting worse, wearable technologies like electrocardiographic (ECG) monitors can be used to continuously monitor heart rate. The ECG is a diagnostic method widely used to evaluate the electrical and motor functionality of the cardiovascular system since it records the rhythm and activity of the heart. The authors of a study published in the Journal of Medical Systems examined 17 studies that assessed the implementation of wearable and mobile ECG monitors for patients with HF. The study discovered that by continuously monitoring heart rate, rhythm, and other ECG data, these devices can assist in identifying early HF exacerbation symptoms and enhance patient outcomes [8]. One of the most popular methods for wearable ECG monitoring is adhesive ECG patches. Wearable ECG patches are wireless, smaller in size, more convenient to use, and more comfortable than the conventional wearable Holter monitor [61]. They can be worn comfortably on the skin, making them convenient for long-term monitoring without the need for frequent electrode placement. Some individuals may experience skin irritation or sensitivity due to the adhesive used in ECG patches. This can become bothersome or uncomfortable with prolonged use [62]. Several studies have used ECG monitoring products in heart rate monitoring which can be potentially used in HF monitoring [9, 63, 64], these ECG patch monitors have a high diagnostic yield in detecting and monitoring arrhythmia in patients with HF [9] and have been cleared for usage by the United States Food and Drug Administration (FDA) and can be used in monitoring heart rate in HF. For instance, the VitalPatch wearable sensor was used in the continuous monitoring of heart rate in HF patients and found that could be used for the early detection of cardiorespiratory deterioration [63]. Another study investigated the accuracy and validity of wearable sensors, including VitalConnect’s VitalPatch, for continuous monitoring of vital signs in elderly individuals to detect and prevent deterioration of patients with HF [64]. Similarly, the ZioXT (iRhythm, San Francisco, CA, USA), NUVANT (Corventis, San Jose, CA, USA), and Savvy monitor (Ljubljana, Finžgarjeva, Slovenia) have the potential to improve HF management and reduce healthcare utilization by providing a convenient and reliable method for monitoring heart function [10]. Fig. 3 presents some wearables used in clinical trials and in the monitoring of heart rate in HF management.

In addition to patches, wearable ECG monitors built into clothing are also common. These devices often use capacitive sensing in conjunction with intelligent materials like e-textiles. These e-textile systems are typically thin, stretchable, flexible, and washable and are highly accurate in monitoring these patients’ cardiac function as they provide real-time data [65]. Stretchable and flexible electrocardiograms are becoming increasingly popular for future wearable and inconspicuous ECG monitoring thanks to advancements in innovative material, fabrication, and printing technology.

*(iii) Blood Oxygen Saturation (SpO):*_2_ This shows how much oxygen is present in the red blood cells that are moving around the body. Most healthy people have SpO_2_ levels between 95% and 100%. If the level drops below this threshold, it indicates that immediate medical attention is required because the person’s organs, tissues, and cells aren’t receiving enough oxygen to function normally [66]. The biomarker of SpO_2_ in HF is crucial. Continuously checking SpO_2_ levels can aid medical professionals in spotting the early warning signs of hypoxia and averting potentially fatal complications. A number of research studies have shown that low SpO_2_ levels are linked to higher mortality rates in individuals suffering from HF [11, 12]. According to a 2020 study, people with HF had a higher risk of mortality and hospitalization when their SpO_2_ levels were low [13]. In emergency scenarios like myocardial infarction, pulse oximetry baseline oxygen saturation is essential for determining the diagnosis and extent of HF and may have prognostic implications. If the baseline pulse oximetry oxygen level is less than 93%, the diagnosis may be suggested. Additionally, it has been demonstrated that SpO_2_ monitoring is a good technique for anticipating HF exacerbation as alarms can be set for only when the reading goes below a certain value. Most oximeters’ default alarm setting is 90% or below. A study by Tobushi *et al*. [14], published in 2019 found that changes in SpO_2_ levels were a reliable early indicator of impending HF decompensation. This suggests that early detection of changes in SpO_2_ levels can allow healthcare providers to intervene early and prevent aggravation before they become life-threatening.

**Fig. 4.**
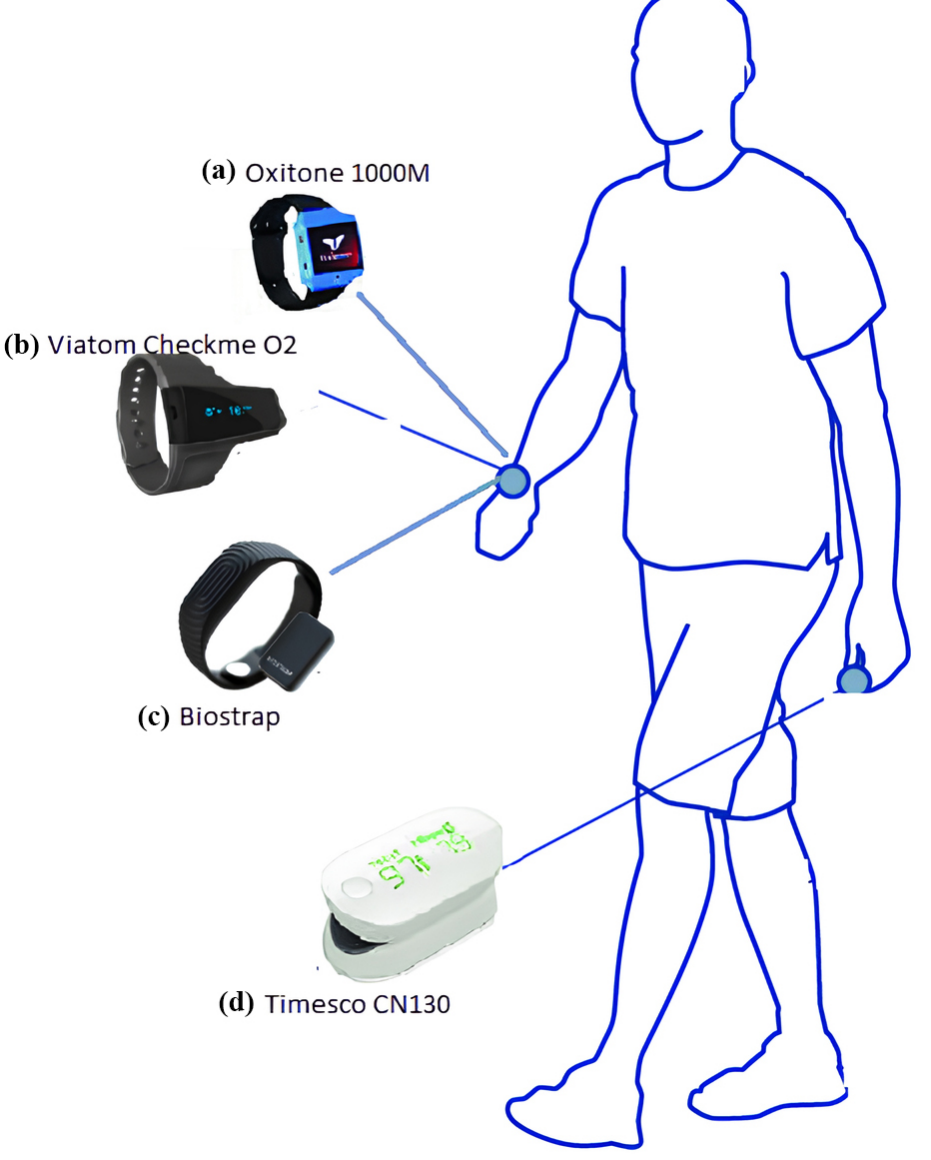
**Wearable pulse oximeters available on the market**. (a) Oxitone 1000M. (b) Viatom Checkme O_2_. (c) Biostrap. (d) Timesco CN130.

In order to identify HF and gauge its severity in emergency scenarios, people with various degrees of HF can reliably utilize a wearable finger pulse oximeter to detect baseline oxygen saturation [15, 67]. Another study discovered that routine cardiac auscultation with the addition of pulse oximetry could be utilized as an accurate and practical early screening for congenital heart disease (CHD) in neonates in widespread clinical practice [68]. Devices for measuring pulse oximetry rely on PPG. A design of many commercially available wearable pulse oximeters is shown in Fig. 4. These devices include SpO_2_ sensors incorporated into them that can detect blood oxygen levels. The built-in detector can identify the various light wavelengths that have travelled through or been reflected from a body part by shining lights from the dual light emitting diode (LED) onto that body part (such as the fingertips or earlobe). The most popular form of this wearable item is a band or wristwatch. The Oxitone 1000M [16] and Checkme O_2_ (Viatom, Shenzhen, China) [69] are commercially offered goods. The difference between the two wavelengths of light absorbed by oxygenated hemoglobin (O_2_Hb) and deoxygenated hemoglobin (DOHb) is then used to determine SpO_2_ (HHb) [17].

### 2.4 Remote Monitoring and Provision of Real-time Feedback

The goal of remote patient monitoring is to use health data collected and transmitted remotely to improve outcomes by capturing patient lifestyle behaviors that may change (e.g., sleep, activity), controlling risk factors, and detecting clinical deterioration or changes in health status before they worsen. Wearable gadgets like smartwatches, thermometers, or pulse oximeters are essential alternatives for people with HF who seek hospital-at-home care. These wearables can be linked to remote monitoring systems, giving medical professionals access to real-time information on a patient’s heart rate, physical activity, blood pressure, and other vital signs [18]. This may make it possible for medical professionals to spot changes in a patient’s condition before they worsen and to administer prompt therapies to stop HF from progressing. Additionally, wearable technology can give patients immediate feedback, motivating them to adopt actions that will help them manage their HF, such as raising their physical activity levels, cutting back on their sodium consumption, and taking their medications as directed. This feedback may encourage patients to continue with their self-care, which may enhance their results and quality of life.

**Fig. 5.**
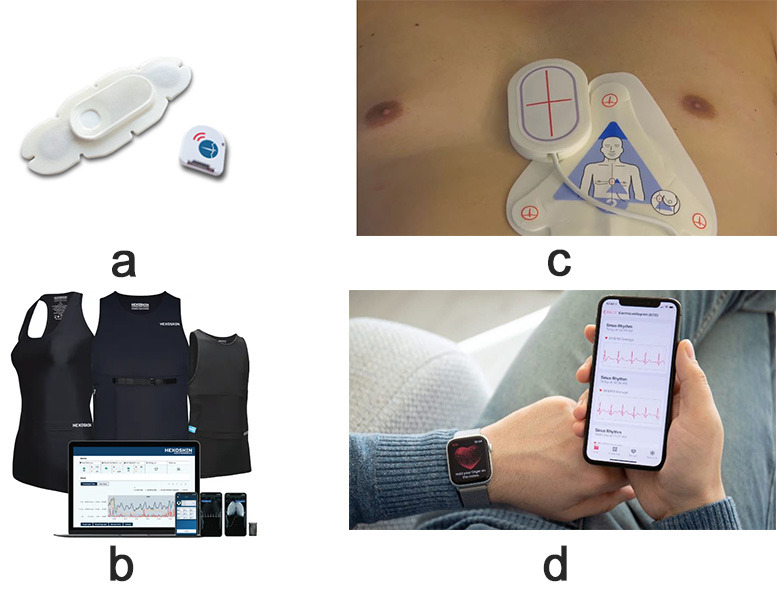
**Non-invasive wearables for remote monitoring and provision of real-time feedback**. (a) Multi-sensor monitoring device with a disposable sensor patch with a disposable battery and a recyclable sensor electronics module. (b) Hexoskin garment. (c) Illustration of the µCor device in the side location of a subject. (d) Wrist-worn sensors.

Several wearables as shown in Fig. 5 can be used for remote monitoring and real-time feedback in heart failure monitoring and management, such as the Hexoskin smart shirt [70], or the ZOLL µCor™ (Microcor) [71], which are wrist-worn sensors [72]. These devices show high accuracy in monitoring patients’ cardiac functions as it provides real-time data through remote monitoring [73]. Multivariate physiological telemetry via a wearable sensor can enable accurate early detection of impending rehospitalization with a prediction accuracy comparable to implanted devices. With 76% to 88% sensitivity and 85% specificity, the platform could identify indicators that a patient would need to be hospitalized for HF exacerbation. This was discovered in a study that looked at how well noninvasive remote monitoring may foretell HF rehospitalization. The median delay between the initial alert and readmission was 6.5 (4.2–13.7) days [74]. These devices for remote monitoring have been proven to reduce re-hospitalization and have appeared feasible for HF medication escalation in HF patients [19, 75].

### 2.5 Tracking Physical Activities

The fourth most common cause of mortality worldwide is inactivity [76]. Reduced cardiovascular morbidity and mortality are linked to even moderate improvements in physical activity (PA), across the general population and in people with pre-existing heart conditions [20, 77]. Patients with HF can benefit greatly from regular exercise. Regular exercise will strengthen the heart and circulatory system and reduce risk factors for heart disease and future heart problems. Although regular exercise and physical activity are promoted for boosting overall cardiovascular health [78]. The current HF guideline does not place enough emphasis on the value of, and recommendations for, physical activity as a method to mitigate the condition [1]. Empirical study [21] has found a correlation between a lower risk of HF and increased physical activity, reduced inactivity, and higher cardiorespiratory fitness. Physical activity may decrease the progression of the illness by lowering the prevalence of HF risk factors, promoting physiological cardiac remodeling, and enhancing mortality and HF symptoms in people who already have the condition [21].

The Apple Watch detects heart rate during exercise with clinically acceptable accuracy in people with cardiovascular disease [79]. The Hannover Medical School in Germany employed the Garmin smartwatch in research that found telemonitoring of exercise to be useful in treating patients with HF [80]. The Chest strap is also a wearable device that can be adopted in monitoring physical activities in individuals with HF [22], chest straps can offer precise heart rate readings, which are important for measuring the level and duration of physical activity. This data can be used to track progress towards activity goals and keep an eye on physical activity levels [23], however, chest straps are typically less convenient and more uncomfortable to wear than other types of wearable devices such as fitness trackers or smartwatches. Therefore, they may not be ideal for long-term monitoring and adherence to wearing them may be a challenge for some individuals. Overall, the choice of a wearable device for physical activity monitoring in HF patients should be based on a variety of factors including accuracy, convenience, comfort, and patient preference [22]. Zhang *et al*. [24], undertook a study to evaluate patients with HF’s physical activity and sleep using Fitbits, and to look into the correlation between these parameters and clinical outcomes. Higher rates of hospitalization and mortality were shown to be associated with lower levels of physical activity and poorer sleep quality, according to the study [24]. Some other wearables which can be used to monitor HF patients during physical activities are still under trial by the FDA, for instance, The TARGET-HF-DM (Innovations to Enhance Effective Adherence and Strengthen Guideline-based Activity Targets in Patients with HF and Diabetes Mellitus) trial which is still ongoing, is trying to assess a digital program aimed at promoting physical activity and medication adherence in individuals with both HF and diabetes [81].

### 2.6 Wearables for Early Detection of Decompensation

The need for immediate hospitalization of HF patients is a critical event, and in-hospital mortality rates range from 4 to 10% [25], and the average cost of admission which varies depending on the health care system is projected to be £2274 in the UK [26] and $14,631 in the USA [27], early detection of decompensation, characterized by the worsening of symptoms and the onset of acute events, is therefore of paramount importance in the management of HF. Timely intervention during these critical periods can prevent disease progression, reduce hospitalization, and improve outcomes.

Wearable technology plays a pivotal role in this early identification process by continuously monitoring key physiological indicators that may signify deterioration in HF patients. For instance, heart rate variability (HRV) is an important indicator that provides insights into the control of the autonomic nervous system over the heart. A decrease in HRV can be an early sign of HF exacerbation. Wearables that track HRV can alert patients and healthcare providers to potential issues before they become critical, aided by smart algorithms that analyze data patterns to enhance the predictive accuracy of potential health declines. Studies, such as one by Li *et al*. [82], highlight how HRV monitoring through wearable devices can predict hospitalization in HF patients due to worsening conditions.

Additionally, bioimpedance analysis helps in measuring fluid retention, a common issue in worsening HF. Wearable devices equipped with sensors to measure bioimpedance can detect increases in fluid accumulation, providing an early warning of HF exacerbation. Groenendaal *et al*. [83] (2021) discuss the use of wearable bioimpedance devices in detecting early signs of fluid accumulation in HF patients, potentially reducing hospital readmissions. Wearable vests that measure intrathoracic impedance have shown a good correlation with fluid status [28, 84]. Intrathoracic impedance can be a biomarker for pulmonary congestion and impending decompensation [85]. In an observational analysis of 91 patients, a non-invasive intrathoracic impedance algorithm achieved a sensitivity of 60% and a specificity of 96% for predicting HF decompensation [86]. A wearable vest, known as Remote Dielectric Sensing (ReDSTM), is currently under research for HF management. An observational study involving 50 patients showed an 87% reduction in hospitalizations with ReDSTM-directed medical titration, compared to the 90 days prior to enrollment; hospitalizations increased by 79% in the 90 days following the removal of the vest [29]. Many clinical trials are ongoing for wearable devices used in monitoring and predicting HF decompensation, some of these wearables include an FDA-approved patch called ZOLL CorTM that measures pulmonary fluid levels and has an ECG monitor, radiofrequency sensor, and transmitter that is currently being tested in a clinical trial for its ability to foretell HF decompensation (NCT03476187, https://clinicaltrials.gov/study/NCT03476187). Also the ability of textile-based sensors to anticipate HF decompensation is now being studied (NCT03719079, https://clinicaltrials.gov/study/NCT03719079).

Moreover, respiratory metrics can indicate cardiovascular stress and fluid overload, which are critical in HF management. Wearables that continuously monitor breathing rates and patterns can alert patients to changes that may indicate a worsening condition [87]. Wearables can capture abnormal respiratory patterns and changes in pulmonary fluid status, aiding in the early detection of HF exacerbations [88]. This proactive approach allows for timely interventions, potentially preventing hospitalizations and improving patient outcomes. The use of wearables for respiratory monitoring complements traditional sporadic measures, offering a more comprehensive view of a patient’s health.

Physical activity levels and energy expenditure are also crucial as reductions in usual activity levels can indicate a decline in heart health. Wearables track the intensity and amount of physical activity, helping to notice deviations from baseline levels that might signify worsening HF [89]. Studies have shown that wearable devices that monitor physical activity can provide early indications of decline in patients with HF, aiding in timely medical intervention.

By incorporating these monitoring functions, wearable devices offer significant advantages in the proactive management of HF. They allow for the early detection of key physiological changes, potentially preventing hospitalizations and improving patient outcomes. Engaging patients in their care process through real-time data also supports better compliance and health management strategies, crucial for managing chronic conditions like HF. This approach not only enhances patient care but also integrates modern technology effectively into everyday health management, ensuring that both patients and healthcare providers can act quickly on potential health issues.

## 3. Barriers/Challenges and Future of Wearables in HF Management 

The integration of wearables in HF management represents a shift from episodic care to continuous, patient-centric monitoring. However, the widespread and effective adoption of wearables faces various barriers and implementation challenges that need to be addressed. Some identified barriers leading to initiative failures include loss of interest, temporary misplacement of the wearable device, concerns about accuracy, financial challenges, and pricing.

The cost implications associated with the acquisition and maintenance of wearable devices may pose a barrier to widespread adoption. While the use of novel technology has been linked to a decrease in mortality and associated expenses due to increased clinic visits, it also comes with an overall rise in costs [90]. Integrating wearable devices into HF management involves initial costs such as device purchase, system integration, and training [91]. However, the long-term benefits include potential savings and a promising return on investment [92]. Wearable technology facilitates early detection and management of HF symptoms, reducing hospital readmissions—studies, including one by the American Heart Association, show up to a 30% reduction in readmissions, which directly lowers healthcare costs [93]. Further, a Price waterhouse and Coopers (PwC) report suggests that widespread adoption of wearable health technologies could save the U.S. healthcare system $200 billion over 25 years by minimizing chronic disease progression [94]. This balance of upfront investment against substantial healthcare savings and improved patient outcomes makes wearable devices a cost-effective solution in managing HF.

Addressing the challenges associated with the clinical implementation of wearable devices in HF management requires detailed consideration of healthcare provider training, workflow integration, and patient acceptance. Effective training is crucial because the effectiveness of wearable technology depends not only on the accuracy of the data collected but also on the ability of clinicians to analyze and act upon this information [92]. Comprehensive training programs should cover the technical operation of devices, the implications of the data collected, and strategies for incorporating this data into patient care plans. Furthermore, integrating these devices into existing healthcare workflows is vital to maintain seamless operations. This involves aligning the device functionality with current medical protocols and ensuring that the data integration does not disrupt existing healthcare services but rather enhances decision-making processes.

When a patient invests in a wearable device, but their physician lacks the infrastructure to receive the data, it creates a potential disconnect between patients and healthcare providers. This gap in connectivity can impede the monitoring and management of the patient’s health, leading to missed opportunities for early intervention. Timely insights into changes in a patient’s health status may be lacking, hindering the realization of the full benefits of remote monitoring and real-time data tracking offered by wearable devices. The inability to seamlessly integrate wearable device data into electronic health records and link it to existing medical records not only limits healthcare providers’ ability to extract valuable insights but also hampers their capacity to make informed decisions about the patient’s care [95]. Moreover, this deficiency in infrastructure may contribute to health inequity, as some patients may have access to advanced monitoring technologies while others do not, resulting in disparities in healthcare access and quality [96]. However, in scenarios where there are facilities to receive these data, the need for standardization of this data becomes paramount, enabling the aggregation, analysis, and utilization of data from diverse sources [97, 98]. It ensures that information exchanged between systems is uniformly understood, facilitating accurate patient assessments and effective treatment plans. By normalizing information into reference terminologies, standardization allows for seamless data integration across the health ecosystem. This process translates data into unique representations, enhancing interoperability and enabling consistent methods for evaluating patient reports. Additionally, standardization aids in the cleaning, qualification, and harmonization of data, improving personalized risk assessments and recommendations. In the context of wearable devices for HF management, such standardization ensures that data from different devices can be integrated effectively into electronic health records, supporting real-time monitoring and decision-making. To reduce errors and improve patient care, technologies and protocols such as Health Level Seven (HL7) and Fast Healthcare Interoperability Resources (FHIR) standards are crucial. These standards facilitate interoperability between different healthcare systems and devices, ensuring that data from wearables can be accurately and efficiently integrated and used in patient management. This is critical in an era dominated by advanced technologies and large data volumes, where standardized procedures and quality management are essential for integrating complex datasets and advancing biomedical knowledge.

The adoption of wearable devices raises concerns about data safety and confidentiality, particularly in the context of protecting patient health information from personal data breaches and unauthorized disclosure. This concern was exemplified by the acquisition of Fitbit by Google [99], sparking worries about the use of personal and health data by a tech giant engaged in AdTech and data commercialization [100]. An assessment of numerous wellness and fitness applications revealed that 74% of apps collected “vital” information and shared it with third parties, with the majority lacking a privacy statement [101]. Wearables with internet access are susceptible to compromise, posing a challenge in designing wearables with security in mind [102, 103]. Wearable device companies prioritize protecting sensitive health data by employing robust encryption methods and adhering to stringent privacy regulations [104, 105]. These companies utilize advanced cryptographic techniques to encrypt data both in transit and at rest, ensuring its security and integrity [106]. Moreover, to comply with regulations like the General Data Protection Regulation (GDPR) in Europe, companies implement measures to handle data responsibly and grant patients extensive rights over their information [107]. By incorporating technologies like Blockchain, Non-Fungible Tokens (NFTs), including encryption, authentication, cloud storage and Trusted Execution Environments (TEE), wearable device companies aim to securely monetize and share data while maintaining privacy and data integrity [108].

Variability in sensor quality across different brands and models can lead to inconsistent results, impacting clinical decision-making and patient management [109]. Studies as [110] highlights the importance of accurate sensors for medical decision support, emphasizing the need for validation studies to quantify and minimize uncertainties in sensor measurements. The lack of validation for wearables poses a substantial hurdle to their effective integration into healthcare, raising questions about the accuracy and reliability of the collected data. In the realm of HF treatment, where precise data is pivotal, the accuracy of wearable-generated data becomes a critical factor for delivering effective care [111]. This concern becomes particularly pronounced in HF management, where reliable physiological data is paramount for informed decision-making in patient care. Validated wearables not only secure the accuracy of information but also enhance the credibility of healthcare interventions and outcomes. Without validation, there exists a risk of inaccurate readings, potential misinterpretation of patient conditions, and compromised patient safety. Assessments of mobile technology for HF patients emphasize the imperative for comprehensive evaluation and validation of such technologies [112]. Validated wearable devices can play a crucial role in determining eligibility for specific HF therapies, emphasizing their accuracy in clinical decision-making [113]. For healthcare providers to make informed decisions about the use of wearables in clinical practice and enhance the quality of care for HF patients, addressing the validation issue is paramount [114].

Patient acceptance and consistency in the use of wearable technology are equally critical to the success of wearable technologies in managing HF [115]. Issues such as discomfort, annoyance, or inconvenience, especially if the devices are bulky or require frequent adjustments, may affect user experience [116]. Regular wear can be hindered by difficulties in device usage, impacting data collection and adherence to monitoring standards [117]. Certain populations, particularly the elderly or those less familiar with digital devices, may resist adopting new technologies, affecting wearable technology adoption and usage [118]. Recent advances in wearable health monitoring devices are tailored for the elderly and offer features like large displays, simple navigation, and voice activation to aid users with reduced dexterity or impaired vision [119]. These devices also enable remote monitoring, facilitating healthcare providers in assisting patients from a distance, which can be particularly beneficial for individuals facing challenges with new technology [120]. Research highlights that wearable technology can enhance physical activity among older adults, with users more likely to meet recommended activity levels compared to non-users [121]. Additionally, the design of wearable articulated manipulators aims to assist elderly individuals with weak hand strength, ensuring stability and safety in grasping objects for daily activities [122]. Such innovations cater to the specific needs of the elderly population, promoting independence and well-being. To enhance user acceptance, wearable technology should prioritize comfort, aesthetics, and utility in its design. Lightweight, ergonomic designs that prioritize user comfort and minimize discomfort can promote long-term wearability and compliance [123]. Educating users on the purpose of wearables in HF management, their potential impact on health outcomes, and how to use and interpret the collected data can further enhance user acceptance and compliance.

Even though continuous health monitoring through wearable devices offers numerous benefits, such as early detection of health issues and improved overall health management, there is a growing concern about the potential risks and disadvantages. The constant flow of information can have unintended consequences on patient psychology and behavior, notably in the form of increased anxiety and inappropriate behavioral changes. This phenomenon, often referred to as “cyberchondria”, involves excessive worrying about one’s health based on the data provided by wearable devices [124], minor fluctuations in heart rate or other vital signs that are within a normal range could be perceived as indicators of serious health issues leading to unnecessary stress and anxiety [125]. To address this issue, it is crucial to develop privacy-aware models that balance the benefits of continuous monitoring with the need to protect user well-being and privacy [126]. By promoting the balanced use and interpretation of health data, and by providing appropriate support and guidance, the phenomenon of cyberchondria can be mitigated, allowing individuals to benefit from continuous health monitoring without unnecessary stress and anxiety.

Wearable devices play a significant role in influencing patient behavior by providing instant feedback on their health status. This feedback can motivate positive health behaviors, such as increasing physical activity [127]. However, there is a concern that patients may misinterpret the data, leading to overcorrection or inappropriate health practices [128] leading to self-diagnosis and self-medication [129]. This can be particularly dangerous if patients adjust their medication doses or change their treatment regimens based on these misinterpretations. There is also a risk that patients may prioritize insights from wearable devices over professional medical advice, potentially leading to non-compliance with prescribed treatments or protocols [130]. To mitigate risks, it is crucial for patients to interpret wearable data in conjunction with professional advice to avoid self-diagnosis, self-medication, and non-compliance with prescribed treatments [131].

There is a risk that both patients and healthcare providers may develop an overreliance on technological solutions for managing health conditions. This dependency can diminish the importance of traditional healthcare practices that are equally or more effective [132]. This overdependency also becomes problematic when technical issues arise—such as battery failure, data loss, or inaccurate readings—which can disrupt patient care and monitoring [128].

Future advancements in wearable technology may include more features for patient and healthcare provider communication and collaboration in order to ensure that patients are receiving accurate and evidence-based guidance, addressing the potential negative effects of wearables on health anxiety and self-management. Furthermore, a greater emphasis might be placed on incorporating wearables into a holistic healthcare management strategy that includes frequent check-ins and professional consultations. Minimizing the hazards associated with self-diagnosis and self-medication would guarantee that patients are receiving advice and assistance that is suited to their unique requirements.

## 4. Conclusions

Wearable health technology is increasingly recognized as a pivotal tool in managing HF, providing significant benefits for both healthcare providers and patients. These devices not only facilitate continuous health monitoring but also promote patient empowerment and engagement by encouraging positive behavioral changes, even in those not achieving complete self-management. However, the transformative potential of wearables encounters limitations due to various factors including technical challenges and privacy concerns. Effective integration of consumer wearables into healthcare systems thus requires strong support from healthcare professionals and active user feedback.

Moving forward, it is crucial for future research to address the barriers identified in this review and to explore the long-term impacts of wearable technology. This entails conducting larger-scale studies to supplement the existing literature, which has primarily focused on smaller investigations. Additionally, there is a need for a greater focus on the user experience and empowerment aspects of wearable technology, an area currently underrepresented in research due to the relatively recent introduction of wearables into the healthcare sector.

The gradual adoption of wearable technology in healthcare presents challenges but also opportunities for strategic advancements. Effective communication with all stakeholders is essential to highlight the long-term benefits of wearable technology. By fostering a more engaged user base, wearables can enable patients to take a more active role in managing their health, potentially reducing the burden on healthcare providers and the system at large. As wearable technology continues to evolve, research must keep pace with these advancements to fully understand and leverage its potential in improving HF care.
